# Factors associated with the prevalence of viral hepatitis B and C among prisoners: Results of two consecutive national surveys in Montenegro

**DOI:** 10.1371/journal.pone.0321464

**Published:** 2025-04-11

**Authors:** Marijan Bakić, Jasmina Stevanović, Marija Milić, Igor Galić, Marija Bakić, Adis Martinović, Nikola Bakić, Mirjana Štrbac, Boško Bakić, Radmila Balaban, Milorad Vujnić, Bojan Joksimović

**Affiliations:** 1 Department of Epidemiology, Institute of Public Health of Montenegro, Podgorica, Montenegro; 2 Department of Epidemiology, Faculty of Medicine, University of Pristina temporarily settled in Kosovska Mitrovica, Kosovska Mitrovica, Serbia; 3 Department of Epidemiology, Community Health Center Bar, Bar, Montenegro; 4 Department of Dermatovenerology, Clinical Center Berane, Berane, Montenegro; 5 Department of Information Technology and Systems, Institute of Public Health of Montenegro, Podgorica, Montenegro; 6 Department of Epidemiology, Insitute of Public Health of Vojvodina, Novi Sad, Serbia; 7 Boško Bakić, Clinic “Moj doktor”, Nikšić, Montenegro; 8 Department of preclinical subjects, Faculty of Medicine Foča, University of East Sarajevo, Republic of Srpska, Foča, Bosnia and Herzegovina; 9 Department of pathophysiology, Faculty of Medicine Banjaluka, University of Banjaluka, Republic of Srpska, Foča, Bosnia and Herzegovina; Centers for Disease Control and Prevention, UNITED STATES OF AMERICA

## Abstract

**Introduction:**

Prisoners are at a higher risk of communicable diseases (such as HIV and hepatitis) than the general population. Therefore, medical screening is crucial for early diagnosis, treatment, identifying those at higher risk of infection, and prevention of infection spread.

**Objective:**

The main objective of this study was to analyze the factors associated with hepatitis B and C seropositivity in the prison population in Montenegro in two consecutive study years.

**Method:**

Prisoners of Prison for Short and Long prison terms in Spuž, Montenegro, were included in two cross-sectional studies during 2012 and 2021. Data on socio-demographic factors, risky behavior, and preventive measures related to blood-borne viruses were collected. The data were statistically processed by statistical testing of differences and applying regression models in SPSS Windows, version 19.

**Results:**

A total of 506 prisoners (2012–298; 2021–208) were included in this study. One fifth of prisoners were seropositive for viral hepatitis B (2012–0.7%; 2021–3.4%) or C (2012–21.8%; 2021-20.7%) or both (2012–0.33%; 2021–0.0%). Factors associated with viral hepatitis B and C seropositivity in both years were shorter prison terms served and injection drug use. Additionally, factors associated with prisoners seropositivity in 2012 were ever drug use and lack of free hygiene kits in prison, and in 2021 were condom use with a permanent partner, non-condom use with non-permanent partner, and availability of free syringes and needles distribution.

**Conclusion and recommendation:**

Prevalent risky behaviors and the lack of harm reduction interventions are more common among seropositive prisoners. Establishing a prison hospital, improving the surveillance system, introducing new or improving old harm reduction interventions is imperative.

## Introduction

There were about 11.5 million people in prisons globally, out of which approximately 0.5 million were imprisoned in Europe [1]. The number of prisoners around the world increased by about 24% between 2000 and 2018, resulting consequently in increase of the number of people working in or visiting prisons, and moving between communities and prisons to approximately 30 million [1–3]. Because of the complex environment that is difficult to control, prisons are ideal places for the transmission of infectious diseases among inmates and are a reservoir for the spread of infections in the community [4]. Overcrowding, increased drug use, tattooing and piercings, sexual violence and other high-risk sexual behaviors, are common in these settings [1,5]. Because drug use and possession are illegal, there is an overrepresentation of drug users in many prison populations [1].

Incarcerated people are more likely to be infected with human immunodeficiency viruses (HIV), hepatitis B (HBV), hepatitis C (HCV), syphilis, gonorrhea, chlamydia, and tuberculosis than members of the general public [6,7]. At any given time, approximately 15% of people in prisons and other closed institutions have HCV, and around 5% have chronic HBV or are living with HIV [6]. Although significantly different from each other, HCV prevalence among prisoners ranged from 4.3–86.3%, among people who inject drugs (PWID) from 13.8–84.3%, and among men who have sex with men (MSM) from 0–4.7% which is significantly higher than the prevalence in the general population [8]. Globally, prisoners who inject drugs have a 6-fold higher prevalence of HIV, 8-fold higher prevalence of HCV and a 2-fold higher prevalence of HBV compared to the non-injecting prisoners [7].

Sustainable Development Goal 3, which is also included in the 2016 The Joint United Nations Programme on HIV and AIDS (UNAIDS) and World Health Organization (WHO) strategies to eliminate acquired immune deficiency syndrome (AIDS) and hepatitis by 2030, can be achieved by prioritizing the prevention, diagnosis and treatment of these infections in key populations [9,10]. The availability of harm reduction interventions in European prisons is limited compared to the community, particularly due to poor implementation [11]. By increasing harm reduction in prison and through care, significant individual and public health benefits can be achieved [11]. During 2022, the WHO published new guidelines for the prevention, diagnosis and treatment of HIV and viral hepatitis in five key populations at increased risk, including prisoners [9].

The prevalence of HIV and viral hepatitis among prisoners was not known in Montenegro until 2012 [12]. In two cross-sectional studies conducted in 2012 and 2021, it was recorded that about a fifth of prisoners have hepatitis C virus infection, about 2% hepatitis B, while HIV infection was not registered [12]. It is interesting that the frequency of these infections in prisons in Montenegro has not changed significantly over a ten-year period [12]. Therefore, the aim of our study is to analyze the factors associated with hepatitis B and C seropositivity in the prison population in Montenegro in two consecutive study years.

## Materials and methods

### Study design and setting

For the purposes of this research, data from two national surveys from March 2012 and December 2021 were used, which were designed as a cross-sectional study and conducted according to the same methodology using the same questionnaires. All prisoners who, during the research period, were serving prison terms in the Prison for Long prison terms and the Prison for Short prison terms in the Department in Spuž were invited to participate in the research. Prisoners sentenced for up to six months are placed in a Prison for Short prison terms, meanwhile anyone over six months are placed in a Prison for Long prison terms.

Namely, in Montenegro, there is one prison institution “Administration for the Execution of Criminal Sanctions”, which has two units, one in the city of Spuž and one in the city of Bijelo Polje. Prisoners from the Prison for Short prison terms in Bijelo Polje were not included in the research due to the small number of prisoners who met the study inclusion criteria, so that completely representative results were obtained for this group of prisoners from the Department in Spuž.

Convicted persons sent to prison are housed in pavilions categorized by security level, in multi-bed rooms. Men and women, minors, foreigners, previously unconvicted from convicted persons, younger from older convicts, and returnees are separated. Prisoners communicate by letter and telephone with family members, legal advisors and receive visits. In the event of the death or serious illness of a close relative, a prisoner, accompanied by a guard, may visit the relative or attend the funeral. Prisoners have the opportunity to regularly inform themselves about public events through the media, and they are allowed to get a job if they wish and receive financial compensation for their work. Health care for all prisoners is organized through the Health Service of the Institution, and in addition to primary health care, they are also provided with dental care and psychiatric care. In case of need for more complex diagnostics and treatment, examinations and medical care of convicted persons are organized in secondary and tertiary healthcare institutions, accompanied by guards and under defined conditions. Security in the Institution is ensured by a system of special measures, and upon admission, the mental health of each prisoner is assessed, whether they pose a danger to other prisoners, staff and visitors, and whether there is a likelihood of self-harm [13].

### Participants

The criteria for inclusion in the study were: age over 18 years, given that interviews with minors under the age of 18 require parents/guardians consent and that they are placed in a special juvenile detention center, as well as prison sentence longer than three months. Criteria for exclusion from the study were: refusal to participate in research, inability to read and write, mental incapacity (inability to understand questions, answer to them and understanding basic ethical principles according to the assessment of the psychiatrist in charge). The survey was anonymous and informed written consent was requested from all prisoners to participate in the research.

In both surveys, an adequate sample size of 255 and 186 prisoners, respectively, was achieved, calculated for a confidence level of 95%, an alpha error of 5%, and the total number of prisoners in 2012 (755) and 2021 (358). Both studies used similar sampling strategies and assumptions to replicate the findings of the previous study, increasing the reliability and generalizability of the study results. Specifically, we aimed to assess changes in the prevalence and patterns of HIV and hepatitis B and C infections in the study population, as well as known risk factors over time, the degree of adoption of standard preventive measures and practices in the prison population, and to identify potential barriers or incentives to their adoption, such as condom use and availability, free distribution of syringes and needles in prison, and a methadone program.

### Ethics statements

The consent of the ethical commission and the director of the Institute for Public Health of Montenegro (number 01 8528 of 19 September 2022) was obtained for the use of data from the aforementioned two consecutive studies in the target population of prisoners. Written informed consent was obtained from all subjects involved in the study.

### Research instruments

The questionnaire was used to collect sociodemographic data, data on risk behaviors and prevention measures related to viral hepatitis B and C and other related infections. The questionnaire was specially created for this purpose based on experience and recommendations obtained from similar research, which was then adapted to the national context of Montenegro. In order to obtain data on seropositivity for HBV and HCV a blood sample was taken from the prisoners.

The organization of the field research was carried out in three phases:

• Phase I - training of all participants in the research
• Phase II - social mobilization which included a conversation with “responsible prisoners” or “leaders” and the organization of the presentation of the research methods to as many prisoners as possible in all parts of the prison, respecting the current epidemiological situation
• Phase III - conducting the survey and blood sampling.

Data collection and blood sampling were performed by qualified persons from the Institute for Public Health of Montenegro and the non-governmental organization Juventas during 2012 and 2021. The research team consisted of interviewers, laboratory technicians/technicians who sampled blood for laboratory analysis, and site coordinators for data entry and control, who were previously trained and prepared to conduct this study. Due to the specifics of the prison environment (mandatory presence of security personnel), the concept of confidentiality required that the data in the questionnaire be collected by self-completion by the prisoner, after an introductory explanation received from the field researcher (interviewer). The researchers in the field supervised the filling of the questionnaires and were available to answer any additional questions the prisoners had regarding the research, as well as any concerns they had regarding the questionnaire, avoiding suggesting answers. Filling out the questionnaire took a maximum of 45 minutes.

Data on socio-demographic, risky behavior, and preventive measures were collected. The questionnaires include questions directly relevant to individual indicators in accordance with international recommendations, the research protocol, and the National Plan for Monitoring and Evaluation of HIV Infections and Hepatitis in Montenegro [14,15].

Biological material from prisoners was collected by venipuncture. Microbiological analyzes of blood samples were performed at the Center for Medical Microbiology of the Institute of Public Health of Montenegro and included testing for the presence of anti-HCV antibodies using an ELISA test (HCV Ab, DIA.PRO Diagnostic Bioprobes Srl, Italy) and testing for the presence of HBs antigen using an ELISA test (HBsAg single version ULTRA, DIA.PRODiagnostic Bioprobes Srl, Italy).

Additional testing of serologically positive persons for hepatitis B and C in order to distinguish acute, chronic or cured infection was carried out only in 2021, given that in 2012 there were no staffing, infrastructure and financial opportunities for that. Naimly, during 2021 in case of samples with a positive finding of HBs antigen, in order to determine acute infection, serological analyzes were performed, testing the presence of anti-HBs antibodies and anti-HBc antibodies of the IgM class using ELISA tests (VIDAS Anti-HBs Total II and VIDAS HBc IgMII, Biomerieux, France) and tests for the presence of total anti-HBc antibodies by ELISA (HBc Ab, DIA.PRO Diagnostic Bioprobes Srl, Italy). In samples with positive anti-HCV antibodies, HCV RNA was determined in serum to determine whether the infection was active, chronic, or already cured. The concentration of viral RNA was determined using the RealTime PCR HCV test (Abbott, USA).

After receiving the test results, all prisoners included in this study underwent post-test counseling and education regarding the prevention of HIV and hepatitis B and C. Prisoners who tested positive on serological screening were referred for further diagnostics and treatment decisions to selected infectious disease specialists.

In accordance with the WHO Guidelines for Testing Hepatitis B and C, focused testing of individuals from populations most affected by these infections is recommended, and serological tests for the detection of HBsAg and HCV antibodies are recommended as a screening test [16]. Testing and diagnosis of chronic HBV and HCV infection using serological tests is a gateway to access prevention, care and treatment services. Early identification of a person with chronic HBV or HCV infection allows them to be referred for additional diagnostic procedures to confirm viremic infection, receive necessary care and treatment, and monitor response to treatment. Additionally, testing offers the chance to link to measures to lower transmission, such as hepatitis B vaccine, counseling on risk behaviors, and the distribution of preventative supplies (such sterilized needles and syringes) [16]. According to the purpose of our study, by applying a screening test in a high-risk population of prisoners, we identified those who are or have been exposed to hepatitis B and C viral infections as a group of seropositive individuals in order to determine factors associated with an increased risk of acquiring these infections. We considered those positive for hepatitis B and C as one group because the prevalence of hepatitis B in the population of Montenegro is low due to mandatory vaccination against this infection for the past 20 years. Also, apart from vaccination against hepatitis B, other preventive measures against blood-borne viruses in high-risk populations are nearly the same due to the sharing of risk factors.

### Statical analysis

Data were analyzed in the user package SPSS Windows, version 19. The level of statistical significance was set at p<0.05. The distribution of data is evaluated using the Kolmogorov-Smirnov test. Measures of central tendency, measures of variability, and relative numbers were used to describe the study population. The Kruskal–Wallis-KW χ2 test and the Mann–Whitney U test were used to examine the differences in the investigated variables between seronegative and seropositive prisoners for viral hepatitis B and C. Factors associated with seropositive results for viral hepatitis B and C among prisoners in 2012 and 2021 were examined using logistic regression analysis. Variables that were significant in the univariate regression analysis were included in the logistic regression analysis.

## Results

### Description of the study sample

Out of a total of 755 prisoners in 2012 (729 men and 26 women) who at the time of the research met the criteria for inclusion in the study, 309 of them or 41.0% (293 men and 19 women) accepted to participate in the research. Valid HBV and HCV test results were obtained from 305 prisoners. Additionally, seven prisoners from 2012 were excluded from the analysis due to incomplete questionnaires, so that in the end, 298 prisoners were included in the analysis. Out of a total of 358 prisoners from 2021 (345 men and 13 women) who at the time of the research met the criteria for inclusion in the study, 222 of them or 60.3% (213 men and 9 women) agreed to participate in the research. Valid HBV and HCV test results and completed questionnaires were obtained from 208 prisoners. In both study years, prisoners were tested for HIV, but no HIV-positive prisoners were registered in any of the study years.

Hepatitis C seropositivity was registered in 108 or one fifth (21.3%) of prisoners, 65 (21.8%) in 2012 and 43 (20.7%) in 2021. Hepatitis B seropositivity was registered in 9 (1.8%) of prisoners, 2 (0.7%) in 2012 and 7 (3.4%) in 2021. Only 1 (0.2%) prisoner had co-infection with Hepatitis B and C virus in 2012 (1 or 0.33%).

Out of a total of 506 prisoners included in these studies, 298 (58.9%) were from 2012, and 208 (41.1%) from 2021 (Table 1). In 2012, the research covered 298 prisoners, 94.6% of whom were men. The mean ± standard deviation (SD) age of prisoners in 2012 was 34.38 ± 9.56 years. Significantly more often than seronegative prisoners, seropositive prisoners had completed only primary school (41.2% versus 19.5%), more precisely, they had a lower educational status. Compared to seronegative prisoners, seropositive prisoners were convicted to current prison term for committing multiple criminal offenses at the same time, not just one specific offense (20.6% vs. 10.4%) and have a higher number of previous convictions in their biography. More precisely, they have already been in prison two to four times (38.2% vs. 20.9%) or more than five times (14.7% vs. 5.7%) (Table 1).

**Table 1:** The difference in the socio-demographic characteristics in the population of prisoners in Montenegro depending on the status of seropositivity for viral hepatitis B and C.

Variables		Serological results 2012 year		p	Serological results 2021 year		p
		negative 230 (77.2%)	positive 68 (22.8%)	negative 158 (76.0%)	positive 50 (24.0%)		
Age*	mean±SD	34.67±9.64	33.41±9.27	0.302	**36.92±11.10**	**34.62±9.13**	**0.049**
Gender	male	219 (95.2)	63 (92.6)	0.408	153 (96.8)	48 (96.0)	0.775
	female	11 (4.8)	5 (7.4)		5 (3.2)	2 (4.0)	
Marital status	single	90 (39.1)	35 (51.5)	0.101	38 (24.1)	15 (30.0)	0.295
	married	84 (36.5)	14 (20.6)		49 (31.0)	10 (20.0)	
	cohabitation/ relationship	20 (8.7)	7 (10.3)		46 (29.1)	13 (26.0)	
	divorced/widowed	36 (15.7)	12 (17.6)		25 (15.8)	12 (24.0)	
Number of children*	mean±SD	0.95±1.37	0.78±1.52	0.194	1.34±2.02	1.04±1.12	0.923
Education	without school	14 (6.1)	7 (10.3)	**0.001**	**13 (8.2)**	**10 (20.0)**	**0.001**
	primary school	**45** (**19.5)**	**28** (**41.2)**		**23 (14.6)**	**17 (34.0)**	
	secondary school	**143** (**62.2)**	**27 (39.7)**		**104 (65.8)**	**18 (36.0)**	
	college/university	28 (12.2)	6 (8.8)		18 (11.4)	5 (10.0)	
Employment	unemployed	105 (45.7)	28 (41.2)	0.212	53 (33.5)	13 (26.0)	0.495
	employed	56 (24.3)	25 (36.8)		51 (32.3)	22 (44.0)	
	self-employed	66 (28.7)	14 (20.6)		51 (32.3)	14 (28.0)	
	pensioner	3 (1.3)	1 (1.4)		3 (1.9)	1 (2.0)	
Type of crime against	property	40 (17.4)	16 (23.5)	**0.007**	**30 (19.0)**	**24 (48.0)**	**0.004**
	public order and peace	10 (4.4)	1 (1.4)		15 (9.5)	2 (4.0)	
	life and body	**49 (21.3)**	**4 (5.9)**		**43 (27.2)**	**6 (12.0)**	
	sexual freedom	6 (2.6)	0 (0.0)		5 (3.2)	1 (2.0)	
	marriage and family	0 (0.0)	0 (0.0)		4 (2.5)	3 (6.0)	
	unauthorized production and trafficking of narcotic drugs	56 (24.3)	19 (28.0)		33 (20.9)	10 (20.0)	
	public transport safety	10 (4.4)	0 (0.0)		7 (4.4)	0 (0.0)	
	unmarked	35 (15.2)	14 (20.6)		11 (7.0)	3 (6.0)	
	several different crimes	**24 (10.4)**	**14 (20.6)**		10 (6.3)	1 (2.0)	
Length of prison terms (year)*	mean±SD	5.35±5.84	4.21±3.81	0.744	3.65±3.43	3.93±3.58	0.757
Length of prison terms served (year)*	mean±SD	2.36±2.19	1.96±1.90	0.258	**2.71±3.42**	**1.36±2.23**	**0.005**
Total number of previous prison terms	this is the first time	**128** (**55.6)**	**15** (**22.1)**	**0.001**	**71** (**44.9)**	**12** (**22.0)**	**0.007**
	one before	41 (17.8)	17 (25.0)		14 (8.9)	4 (8.0)	
	two to four times	**48** (**20.9)**	**26** (**38.2)**		**50** (**31.6)**	**29** (**58.0)**	
	five and more times	**13** (**5.7)**	**10** (**14.7)**		23 (14.6)	6 (12.0)	

*Numeric variable; SD-standard deviation

In 2021, the research included 208 prisoners, 96.6% of whom were men. The mean ± standard deviation (SD) age of prisoners in 2021 was 36.37 ± 10.69 years. Also in 2021, significantly more often than seronegative prisoners, seropositive prisoners had completed only primary school (34.0% versus 14.6%), more precisely, they had a lower educational status. Compared to seronegative prisoners, seropositive prisoners from 2021 were in prison for a shorter time (1.36±2.23 vs. 2.71±3.42 years), and previously they resided in prison more often, usually two to four times (58.0% vs. 31.6%) and (Table 1).

### Prisoners’ risky behavior

In survey conducted in 2012, 46.3% of prisoners ever used drugs, 15.8% injected drugs, 5.4% shared drug injection equipment, 51.7% ever got a tattoos. Also in the same year, a significantly higher percentage of prisoners who were seropositive compared to seronegative ever used drugs (77.9% vs. 37.0%) and injected drugs (52.9% vs. 4.8%). Injecting drugs in seropositive prisoners was more often longer than one year (47.1% vs. 3.9%) and they were more often on methadone maintenance therapy (5.9% vs. 0.0%). Initiation of drug injection in seropositive prisoners occurred more often outside the prison (51.5% vs. 4.3%), with the more often use of shared drug injection equipment (23.5% vs. 0.0%). Seropositive prisoners got tattoos more often (67.6% versus 47.0%), and for tattoos they more often used needles whose sterility they were not sure of (33.8% versus 17.8%) (Table 2).

**Table 2:** The difference in risky behavior in the population of prisoners in Montenegro depending on the status of seropositivity for viral hepatitis B and C.

Variables		Serological results 2012 year		p	Serological results 2021 year		p
		negative 230 (77.2%)	positive 68 (22.8%)	negative 158 (76.0%)	positive 50 (24.0%)		
**Ever used drugs**	no	**145 (63.0)**	**15 (22.1)**	**0.001**	**74 (46.8)**	**8 (16.0)**	**0.001**
	yes	**85 (37.0)**	**53 (77.9)**		**84 (53.2)**	**42 (84.0)**	
Age of first ever drug use*	mean±SD	18.81±5.56	17.12±3.99	0.146	18.55±4.03	17.76±4.98	0.091
**The possibility of obtaining drugs in prison**	no	97 (42.2)	31 (45.6)	0.249	**12 (7.6)**	**6 (12.0)**	**0.001**
	yes	40 (17.4)	16 (23.5)		**7 (4.4)**	**19 (38.0)**	
	don’t know	93 (40.4)	21 (30.9)		**139 (88.0)**	**25 (50.0)**	
**Injecting drugs**	no	**219 (95.2)**	**32 (47.1)**	**0.001**	**154 (97.5)**	**18 (36.0)**	**0.001**
	yes	**11 (4.8)**	**36 (52.9)**		**4 (2.5)**	**32 (64.0)**	
Age of first drug injection*	mean±SD	23.00±3.68	20.72±1.52	0.121	20.25±8.77	21.88±7.42	0.479
**Last drug injection**	≤ 1 year	**2 (0.9)**	**4 (5.8)**	**0.001**	**0 (0.0)**	**9 (18.0)**	**0.001**
	> 1 year	**9 (3.9)**	**32 (47.1)**		**4 (2.5)**	**23 (46.0)**	
	not inject drugs	**219 (95.2)**	**32 (47.1)**		**154 (97.5)**	**18 (36.0)**	
**First drug injection in prison**	no	**10 (4.3)**	**35 (51.5)**	**0.001**	**4 (2.5)**	**32 (64.0)**	**0.001**
	yes	1 (0.4)	1 (1.4)		**/**	**/**	
	not inject drugs	**219 (95.3)**	**32 (47.1)**		**154 (97.5)**	**18 (36.0)**	
**Sharing drug injection equipment**	no	**230 (100.0)**	**52 (76.5)**	**0.001**	**157 (99.4)**	**33 (66.0)**	**0.001**
	yes	**0 (0.0)**	**16 (23.5)**		**1 (0.6)**	**17 (34.0)**	
**Methadone maintenance therapy**	no	**230 (100.0)**	**64 (94.1)**	**0.003**	**156 (98.7)**	**34 (68.0)**	**0.001**
	yes	0 (0.0)	4 (5.9)		**2 (1.3)**	**16 (32.0)**	
**Ever get a tattoo**	no	**122 (53.0)**	**22 (32.4)**	**0.004**	**42 (26.6)**	**4 (8.0)**	**0.006**
	yes	**108 (47.0)**	**46 (67.6)**		**116 (73.4)**	**46 (92.0)**	
**Using sterine needles for tattooing**	no	7 (3.1)	5 (7.4)	**0.004**	7 (4.4)	3 (6.0)	**0.012**
	yes	**41 (17.8)**	**23 (33.8)**		**17 (10.8)**	**12 (24.0)**	
	don’t know	60 (26.1)	18 (26.4)		92 (58.2)	31 (62.0)	
	not tattooing	**122 (53.0)**	**22 (32.4)**		**42 (26.6)**	**4 (8.0)**	
**Need to provide sterile tattoo needles in the prison**	no	41 (17.8)	8 (11.8)	0.302	50 (31.6)	12 (24.0)	**0.049**
	yes	84 (36.5)	31 (45.6)		**69 (43.7)**	**31 (62.0)**	
	don’t know	105 (45.7)	29 (42.6)		39 (24.7)	7 (14.0)	

*Numeric variable; SD-standard deviation

In survey conducted in 2021, 60.6% of prisoners ever used drugs, 17.3% injected drugs, 8.7% shared drug injection equipment, 77.9% ever got a tattoos. Significantly higher percentage of prisoners who were seropositive in 2021 reported ever used drugs (84.0% vs. 53.2%) and stated that it was possible to obtain narcotics in prison (38.0% vs. 4.4%). Seropositive prisoners more often injected drugs (64.0% vs. 2.5%) for a period longer than a year (46.0% vs. 2.5%) and were more often on a methadone maintenance therapy (32.0% according to 1.3%). Injecting drugs initiation among seropositive prisoners compared to seronegative ones occurred more often outside the prison (64.0% vs. 2.5%), with the use of shared drug injection equipment (34.0% vs. 0.6%). Compared to seronegative prisoners, seropositive prisoners got tattoos more often (92.0 vs. 73.4%), they more often used needles for tattoos whose sterility they were not sure of (24.0% vs. 10.8%), but they considered that it is necessary to provide them with sterile needles for tattooing in prison (62.0% vs. 43.7%) (Table 2).

In 2012, 5.4% of prisoners had sex with in prison non-permanent partners, 13.42% do not use condom while 39.60% use it periodically when having sex with a non-permanent partner. When it comes to sexual behavior, seropositive compared to seronegative prisoners are significantly less likely to have permanent sexual partners (41.2% compared to 54.3%), less likely to avoid using condoms during sex with a permanent partner (35.3% in compared to 54.8%), but are more likely to occasionally use condoms during sex with a permanent partner (42.6% compared to 29.6%) (Table 3). In 2021, 2.4% of prisoners had sex with non-permanent partners, 8.7% do not use condom while 15.9% use it periodically when having sex with a non-permanent partner. When it comes to sexual behavior, seropositive compared to seronegative prisoners are more likely to not use a condom during sex with not-permanent partner (18.0% compared to 5.7%) (Table 3).

**Table 3:** The difference in risky forms of sexual behavior in the population of prisoners in Montenegro depending on the status of seropositivity for viral hepatitis B and C.

Variables		Serological results 2012 year		p	Serological results 2021 year		p
		negative 230 (77.2%)	positive 68 (22.8%)	negative 158 (76.0%)	positive 50 (24.0%)		
**Sex ever**	no	16 (7.0)	5 (7.4)	0.911	3 (1.9)	0 (0,0)	0.326
	yes	214 (93,0)	63 (92,6)		155 (98.1)	50 (100,0)	
**Sex in prison**	no	192 (83.5)	59 (86.8)	0.575	119 (75.3)	42 (84.0)	0.201
	yes	38 (16.5)	9 (13.2)		39 (24.7)	8 (16.0)	
**Sex with permanent partner**	no	**105 (45,7)**	**40 (58.8)**	**0.050**	65 (41,1)	20 (40.0)	0.886
	yes	**125 (54,3)**	**28 (41.2)**		93 (58,9)	30 (60.0)	
**Sex in prison with non-permanent partner**	no	218 (94,8)	64 (94.1)	0.831	155 (98,1)	47 (94.0)	0.131
	yes	12 (5,2)	4 (5.9)		3 (1,9)	2 (6.0)	
**Number of persons who are not-permanent sexual partners**	mean±SD	7,77±13,58	3,67±4,93	0.718	1,00±0,00	1,00±0,00	1.000
**Sex in prison with a person of the same gender**	no	230 (100,0)	68 (100,0)	/	158 (100,0)	49 (98.0)	0.075
	yes	0 (0.0)	0 (0.0)		0 (0,0)	1 (2,0)	
**Sex in prison with an injecting drug user**	no	226 (98,3)	67 (98,5)	0.858	155 (98.1)	49 (98.0)	0.964
	yes	1 (0,4)	0 (0,0)		3 (1,9)	1 (2,0)	
	don’t know	3 (1,3)	1 (1,5)		/	/	
**Use of condoms with a permanent partner**	no	**126 (54.8)**	**24 (35.4)**	**0.042**	71 (44.9)	23 (46.0)	0.870
	periodically	**68 (29.6)**	**29 (42.6)**		20 (12.7)	7 (14.0)	
	always	12 (5.2)	6 (8.8)		2 (1,3)	0 (0,0)	
	don’t have a partner	24 (10.4)	9 (13.2)		65 (41.1)	20 (40.0)	
**Using a condom with non-permanent partner**	no	28 (12.2)	12 (17.6)	0.433	**9 (5.7)**	**9 (18,0)**	**0.031**
	periodically	91 (39.6)	27 (39.7)		27 (17,1)	6 (12,0)	
	always	35 (15.2)	6 (8,9)		12 (7.6)	6 (12.0)	
	don’t have a partner	76 (33.0)	23 (33.8)		110 (69.6)	29 (58.0)	
**Condom used during the last sexual intercourse**	no	191 (83.0)	57 (83.8)	0.880	136 (86.1)	41 (82.0)	0.481
	yes	39 (17.0)	11 (16.2)		22 (13,9)	9 (18.0)	

*numeric variable; SD-standard deviation

### Preventive measures in prisons

During the 2012 survey, seropositive prisoners indicated that they received significantly fewer free hygiene kits compared to seronegative prisoners (23.5% vs. 40.0%) but more HIV brochures (23.5% vs. 13.0%). However, compared to seronegative prisoners, seropositive prisoners were significantly more often educated about HIV (38.2% vs. 23.9%), sexually transmitted infections (35.4% vs. 20.4%), viral hepatitis (30.9% vs. 20.0%), and addiction (35.4% vs. 25.2%). More seropositive prisoners had ever been tested for HIV, all in prison (35.4% vs. 21.7%), and compared to seronegative prisoners, more of them wanted to be tested than were forced to get tested (44.1% vs. 20.9%) (Table 4).

**Table 4:** The difference in prevention measures in the population of prisoners in Montenegro depending on the status of seropositivity for viral hepatitis B and C.

Variables		Serological results 2012 year		p	Serological results 2021 year		p
		negative 230 (77.2%)	positive 68 (22.8%)	negative 158 (76.0%)	positive 50 (24.0%)		
**Condoms free distribution in prison**	no	221 (96.1)	65 (95.6)	0.854	155 (98.1)	48 (98.0)	0.950
	yes	9 (3.9)	3 (4.4)		3 (1.9)	1 (2.0)	
**Hygiene kits free distribution in prison**	no	**138 (60.0)**	**52 (76.5)**	**0.013**	**79 (50.0)**	**16 (32.0)**	**0.026**
	yes	**92 (40.0)**	**16 (23.5)**		**79 (50.0)**	**34 (68.0)**	
**Books and magazines free distribution in prison**	no	181 (78.7)	55 (80.9)	0.696	95 (61.1)	33 (66.0)	0.457
	yes	549 (21.3)	13 (19.1)		63 (38.9)	17 (34.0)	
**HIV brochures free distribution in prison**	no	**200 (87.0)**	**52 (76.5)**	**0.036**	129 (81.6)	39 (78.0)	0.569
	yes	**30 (13.0)**	**16 (23.5)**		29 (18.4)	11 (22.0)	
**Syringes and needles free distribution in prison**	no	220 (95.7)	65 (95.6)	0.982	157 (99.4)	48 (96.0)	**0.042**
	yes	10 (4.6)	3 (4.4)		**1 (0.6)**	**2 (4.0)**	
**Education about HIV**	no	**175 (76.1)**	**42 (61.8)**	**0.020**	115 (71.5)	34 (66.0)	0.457
	yes	**55 (23.9)**	**26 (38.2)**		43 (28.5)	16 (34.0)	
**Education about sexually transmitted infections**	no	**183 (79.6)**	**44 (64.6)**	**0.012**	126 (72.0)	31 (66.0)	0.513
	yes	**47 (20.4)**	**24 (35.4)**		49 (28.0)	16 (34.0)	
**Education about viral hepatitis**	no	**184 (80.0)**	**47 (9.1)**	**0.049**	113 (71.5)	32 (64.0)	0.313
	yes	**46 (20.0)**	**21 (30.9)**		45 (28.5)	18 (36.0)	
**Addiction education**	no	**172 (74.8)**	**44 (64.6)**	**0.050**	113 (71.5)	31 (62.0)	0.204
	yes	**58 (25.2)**	**24 (35.4)**		45 (28.5)	19 (38.0)	
**Do you know about the HIV virus and the AIDS disease?**	no	29 (12.6)	4 (5.9)	0.120	10 (6.3)	1 (2.0)	0.233
	yes	201 (87.4)	64 (94.1)		148 (93.7)	49 (98.0)	
**Knowledge score** *	mean±SD	10.10±2.49	10.34±2.82	0.785	9.95±2.31	9.98±2.39	0.821
**Do you know where to get tested for HIV?**	no	130 (56.5)	33 (48.5)	0.245	74 (46.8)	19 (38.0)	0.273
	yes	100 (43.5)	35 (51.5)		84 (53.2)	31 (62.0)	
**Have you ever been tested for HIV?**	not	180 (78.3)	44 (64.6)	**0.023**	**101 (62.9)**	**23 (46.0)**	**0.001**
	yes. in prison	**50 (21.7)**	**24 (35.4)**		40 (25.3)	11 (22.0)	
	yes. before prison	/	/		**17 (10.8)**	**16 (32.0)**	
**Have you been notified of your HIV test results?**	no	12 (5.2)	11 (16.2)	0.880	**5 (3.2)**	**5 (10.0)**	**0.029**
	yes	43 (18.7)	19 (27.9)		52 (32.9)	22 (44.0)	
	not tested	175 (76.1)	38 (55.9)		**101 (63.9)**	**23 (46.0)**	
**Reason for testing**	wanted testing	**48 (20.9)**	**30 (44.2)**	**0.001**	**53 (33.5)**	**23 (50.0)**	0.060
	forced testing	**8 (3.5)**	**1 (1.4)**		3 (1.9)	2 (4.0)	
	not tested	**174 (75.7)**	**37 (54.4)**		**102 (64.6)**	**23 (46.0)**	
**Is HIV testing available in prison?**	no	55 (23.9)	22 (32.4)	0.331	22 (13.9)	11 (22.0)	0.395
	yes	99 (43.1)	28 (41.2)		105 (66.5)	30 (60.0)	
	don’t know	76 (33.0)	18 (26.4)		31 (19.6)	9 (18.0)	

*Numeric variable; SD-standard deviation

During the 2021 survey, seropositive compared to seronegative prisoners indicated that they received significantly fewer free hygiene kits (32.0% vs. 50.0%), but more free syringes and needles (4.0% vs. 0.6%), more often report ever HIV testing, mostly outside prison (32.0% vs. 10.8%), were less likely to be notified of test results (10.0% vs. 3.2%). Compared to seronegative prisoners, seropositive prisoners were more likely to state that they wanted to be tested rather than forced to be tested for HIV (50.0% vs. 33.5%) (Table 3).

### Regression models

Logistic regression analyzes examining the association of sociodemographic and behavioral characteristics of prisoners, as well as the implementation of preventive measures in prisons with status of seropositivity for viral hepatitis B and C, revealed significant equations in both study years when successive surveys were conducted (2012 year: B = -1.219; Wald = 77.933; Exp(B) = 0.296; p=0.001 and 2021 year: B = -1.151; Wald = 50.280; Exp(B) = 0.316; p= 0,001).

Factors associated with seropositivity for viral hepatitis B and C among prisoners in 2012 were shorter length of prison terms served, drug use, injecting drugs, and lack of free hygiene kits distribution in prison (Table 5).

**Table 5:** Regression analysis of factors associated with seropositivity for viral hepatitis B and C in the prison population in Montenegro in 2012.

Variables	Socio-demographic model			Behavior model			Entire model		
	Exp(B)	95% CI	p	Exp(B)	95% CI	p	Exp(B)	95% CI	p
Age Education Total number of previous prison terms	1.00	0.97; 1.03	0.910	1.02	0.98; 1.07	0.248	1.03	0.99; 1.07	0.218
	**0.65**	**0.44; 0,95**	**0.028**	**0.63**	**0.39; 0.99**	**0.050**	0.64	0.39; 1.03	0.065
	**1.86**	**1.40; 2,47**	**0.001**	1.30	0.92; 1.85	0.143	1.27	0.88; 1.84	0.203
**Length of prison terms served (year)**	0.88	0.75; 1,03	0.115	0.83	0.69; 1.01	0.056	**0.81**	**0.67; 0.99**	**0.036**
**Ever used drugs**				2.14	0.91; 5.03	0.080	**2.39**	**1.01; 5.84**	**0,049**
The possibility of obtaining drugs in prison				1.10	0.74; 1.64	0.624	1.13	0.75, 1.71	0.555
**Injecting drugs**				**11.76**	**4.72; 29.32**	**0.001**	**11.58**	**4.34; 30.90**	**0.001**
Tattoo ever				1.29	0.62; 2.68	0.504	1.26	0.58; 2.74	0.564
Sex with permanent partner				0.78	0.37; 1.66	0.515	0.65	0,29; 1.44	0.289
Use of condoms with a permanent partner				1.30	0.88; 1.92	0.188	1.20	0.80; 1.78	0.376
Use of condoms with a non-permanent partner				0.92	0.65; 1.32	0.660	0.92	0.64; 1.32	0.645
Have you ever been tested for HIV?							0.91	0.39; 2.16	0.832
Need to provide sterile tattoo needles in the prison							1.11	0.67; 1.86	0.680
**Hygiene kits free distribution in prison**							**0.37**	**0,15; 0.87**	**0.023**
HIV brochures free distribution in prison							1.38	0.42; 4.52	0.595
Syringes and needles free distribution in prison							1.60	0.24; 10.62	0.628
Education about HIV							1.75	0.29; 10.70	0.545
Education about sexually transmitted infections							0.86	0.11; 7.06	0.892
Education about viral hepatitis							1.65	0.37; 7.38	0.514
Addiction education							0.49	0.08; 2.79	0.418
Nagelkerke R Square	0.153			0.421			0.449		
Omnibus Tests of Model Coefficients Sig.	**0.001**			**0.001**			**0.001**		

Factors associated with seropositivity for viral hepatitis B and C among prisoners in 2021 were a shorter length of prison terms served, injecting drug, condom use with a permanent partner, no condom use with a not-permanent partner, and the availability of free distribution of syringes and needles in prison (Table 6).

**Table 6:** Regression analysis of factors associated with seropositivity for viral hepatitis B and C in the prison population in Montenegro in 2021.

Variables	Socio-demographic model			Behavior model			Entire model		
	Exp(B)	95% CI	p	Exp(B)	95% CI	p	Exp(B)	95% CI	p
Age Education Total number of previous prison terms	0.99	0.96; 1.02	0.551	1.02	0.97; 1.07	0.439	1.01	0.95; 1.06	0.809
	**0.57**	**0.37; 0,86**	**0.007**	0.98	0.52; 0.83	0.939	0.92	0.47; 1.81	0.818
	1.32	0.97; 1.81	0.081	0.89	0.55; 1.42	0.612	1.94	0.56; 1.60	0.826
**Length of prison terms served (year)**	**0.86**	**0.73; 1,00**	**0.050**	0.83	0.67; 1.02	0.075	0.82	0.65; 1.05	0.118
**Ever used drugs**				1.20	0.41; 3.55	0.743	1.82	0.51; 6.49	0,351
The possibility of obtaining drugs in prison				1.59	0.63; 3.96	0.324	1.74	0.62, 4.86	0.290
**Injecting drugs**				**127.93**	**23.52; 695.69**	**0.001**	**223.81**	**33.78; 1482.77**	**0.001**
Tattoo ever				0.88	0.22; 3.53	0.857	0.76	0.15; 3.92	0.745
Sex with permanent partner				2.61	0.08; 84.10	0.588	25.53	0,61; 1075.30	0.090
**Use of condoms with a permanent partner**				1.52	0.45; 5.12	0.502	**3,40**	**1.91; 12.55**	**0.047**
**Use of condoms with a non-permanent partner**				**0.60**	**0.37; 0.97**	**0.038**	**0.49**	**0.28; 0.86**	**0.012**
Have you ever been tested for HIV?							1.03	0.51; 2.07	0.938
Need to provide sterile tattoo needles in the prison							0.45	0.20; 1.02	0.055
Hygiene kits free distribution in prison							1.92	0,65; 5.72	0.240
HIV brochures free distribution in prison							1.53	0.27; 8.81	0.635
**Syringes and needles free distribution in prison**							**31.88**	**1.22; 830.71**	**0.037**
Education about HIV							0.32	0.00; 466.81	0.761
Education about sexually transmitted infections							0.07	0.00; 1.65	0.098
Education about viral hepatitis							3.26	0.00; 3096.26	0.735
Addiction education							8.50	0.84; 86.19	0.070
Nagelkerke R Square	0.144			0.566			0.643		
Omnibus Tests of Model Coefficients Sig.	**0.001**			**0.001**			**0.001**		

## Discussion

In both study years the prevalence of HCV and HBV in 2012 was 21.8% and 0.7%, and in 2021 20.7% and 3.4%, respectively. The results of our study showed that shorter length of prison terms served and drug injection were factors consistently associated with hepatitis B and C seropositivity in both study years. Specifically, drug use and the lack of distribution of free hygiene kits in prison were associated with seropositivity in 2012, while in 2021 it was condom use with a permanent partner, non-use of condoms with a non-permanent partner, and the availability of free distribution of syringes and needles in prison. It is interesting that in both study years no HIV was registered among prisoners in Montenegro.

Overall, the prison population has much higher rates of all infections than the general population, especially in areas where HIV and injection drug use is highly prevalent [6,8,17]. According to the results of a German study, prisoners have a 48–69 times higher risk of HCV infection and a 7–12 times higher risk of HIV infection compared to the general population [17]. Overall, it was found that in Europe, the subpopulations with the highest prevalence of HBsAg and anti-HCV are prisoners (range: 0.3% - 25.2% and 4.3% - 86.3%, respectively), followed by PWID (0, 5% - 6.1% and 3.8% - 84.3%) and MSM (0.0% - 1.4% and 0.0% - 4.7%) [8]. Compared to our results, seroprevalences for HBV (11.3%), HBV/HCV (6.3%) and HIV (0.15%) were slightly higher in Croatian prisons, but lower for HCV (8.3%) [18]. A similar pattern was observed in the German (HCV-17.6%, HIV-0.8%) and Turkey (HCV- 17.7%, HBV-2.6%) populations [17,19,20].

According to our results, drug use and drug injection, as well as high-risk sexual activities are considered to be the main factors associated with seropositivity on viral hepatitis B and C [6,21]. The percentage of injecting drugs in prison worldwide varies from about 10% to 60% [22–24]. In our study, the prevalence of drug injection was 15% in 2012 and 17% in 2021, which is half less than neighboring Serbia (38%) [25]. Research has shown that about 11% of formerly incarcerated people started injecting drugs while in prison, while about 30% of ex-offenders had done so before [17]. Incarceration has been found to double the odds of restarting intravenous drug use among former intravenous drug users who were not injecting at the time of incarceration [26]. The average prevalence of anti-HCV in the population of intravenous drug users in the neighboring Balkan countries is about 40% and ranges up to 60%, while the average prevalence of HBsAg was in the range of 5–10% [27]. An HBV infection occurred in one-fifth of persons with histories of drug injection in USA, which is more than four times higher than the frequency in the general population [28]. Prisoners with a history of injecting drugs are about 24 times more likely to have HCV infection than those without a history of injecting [21]. A scope analysis that looked at deaths from infections in prisoners worldwide over a 20-year period found that TB, HCV, HIV/AIDS, HIV/TB and HBV was associated with the highest number of deaths [29,30].

North America and Europe have the highest rate of sexual activity among prisoners at 12.1%, while in our study it was 15% in 2012 and 22% in the 2021 research year [31]. More than 34% of Catalan inmates in Spain admitted to having sex in incarcerated [32]. Research shows that men in prison are a significant source of sexually transmitted infections for their sexual partners because they have multiple sexual partners or use sex work as a means of obtaining drugs or money [33]. Condom distribution is free in only one-third of the world’s countries, despite the fact that it is one of the most important interventions for reducing the spread of infection in prisons [34,35]. The long-term effects of providing condoms in prisons increase the likelihood of their use, reduce the prevalence of sexually transmitted infections among prisoners, and do not affect the frequency of sex in prisons [36,37]. In our study, condom use with a permanent partner and non-use of condoms with a non-permanent partner were associated with seropositivity for hepatitis B and C virus infection. The increased use of condoms with a permanent partner can be interpreted as a desire to protect the partner from possible infections of which the prisoners are aware, while non-use or occasional use of condoms with a non-permanent partner indicates a possible route of infection transmission, tendency towards risky behavior and the need for preventive intervention. Also, the association between more frequent condom use during sex with a permanent partner and hepatitis B and C seropositivity in prisoners may be the result of the permanent partner’s insistence on using condoms during sex in prison and its availability, given that the permanent partner is able to buy condoms outside of prison. Additional risky prison behaviors that contribute to the spread of HBV, and HCV infections include body piercing and non-sterile tattooing [31,38]. In our studies, 51% of prisoners had a tattoo in 2012 and 77% in 2021, and about 20% of prisoners in both studies did not use sterile needles or were not sure that the tattoo needles were sterile. High levels of tattooing were recorded in prisons in Latin America (45.4%) and Asia and Pacific (21.4%), but significantly lower in Europe and North America (14.7%) [31]. A study from Puerto Rico shows that about 60% of prisoners acquired tattoos in prison, and that 12% of non-injecting prisoners who received tattoos in prison have HCV. Excluding injection drug use, tattooing with reused needles or sharps and/or ink reuse was 2.6 times more associated with self-reported HCV [39]. Sixty percent of prisoners in Puerto Rico got their tattoos by sharing needles and unsterilized sharp objects which only indicates the importance of prevention of this form of risky behavior [38].

In recent years, institutions around the world have recognized how important it is to treat the health of prisoners as an integral part of public health. However, there are a number of issues and compared to the community, European prisons have limited access to and coverage of harm reduction measures [4,11]. Although lubricant distribution, counselling on safer injecting and tattooing/piercing and needle and syringe programs are rare, opioid substitution therapy (OST) is available in 15% of European prisons, with coverage below 30% in most of them [11]. Testing for infectious diseases is not required on entry to prison and treatment for infections is available, with low coverage for hepatitis B and C and high coverage for HIV and tuberculosis [11]. The lack of distribution of free hygiene kits in the prison is related to the seropositivity of our prisoners in 2012, while in 2021 it was the availability of free distribution of syringes and needles in the prison. Our results indicate that the first study from 2012 pointed out existing problems in prisons in Montenegro and led to the improvement of hygiene and infection control in prisons, as well as increased the availability of free syringes and needles, which was confirmed by a significantly higher number of seropositive prisoners in 2021. In general, prison health care in Montenegro is partly connected to the national health system and for example OST cannot be started in prison, it is only available as a continuation of treatment, and education and harm reduction programs are scarce [40,41].

These studies were the first large-scale investigations in the prison population and allow the analysis of trends in the frequency of risk factors and tested infections, as well as the effectiveness of applied harm reduction interventions. Because all inmate information, including prior drug use, risky sexual activity, and testing experience, was self-reported, and because this study examined a sensitive topic in a sensitive population, it is susceptible to information bias. Due to the cross-sectional study design, the direction of association cannot be defined and causal pathways cannot be inferred.

## Conclusions

It was observed that the prevalence of HBV and HCV has not changed significantly over a ten-year period in prisons in Montenegro and that the frequency of HCV infection is particularly high. Our results showed that drug use, and above all its injection, is the factor most strongly associated with seropositivity for viral hepatitis B and C, which are registered in a fifth of our prisoners. Likewise, the lack of OST therapy and prevalent risky behaviors are more common among seropositive prisoners. Our results indicate that the inadequate use of condoms with non-permanent partners and the unavailability of hygiene kits are associated with seropositivity for viral hepatitis, but also that seropositive prisoners use condoms more often with regular partners and that free syringes and needles for injecting drugs are available to them. Therefore, establishing a prison hospital, improving the surveillance system, introducing new or improving old risk reduction interventions is imperative in our country.
